# Supplementary material for: Dysregulated plasma lipid mediator profiles in critically ill COVID-19 patients
Source: PLoS One. 2021 Aug 26;16(8):e0256226. doi: 10.1371/journal.pone.0256226 (PMC8389414; doi:10.1371/journal.pone.0256226)
Supplement: S1 Table — The table displays the mean values ± sem of LM concentrations in pg/mL from plasma collected from COVID-19 patients with severe disease (n = 15) and critically ill patients (n = 23). (DOCX) [file pone.0256226.s001.docx]

**S1 Table. Distinct plasma LM concentrations in patients with severe disease and critically ill patients.**

| **Group** | **Severe (n=15)** | | | **Critically Ill (n=23)** | | |
| --- | --- | --- | --- | --- | --- | --- |
|  | **mean** | **±** | **sem** | **mean** | **±** | **sem** |
| **DHA Bioactive Metabolome** | | | | | | |
| RvD1 | 1.118 | ± | 0.51 | 0.23 | ± | 0.11 |
| RvD2 | 0.055 | ± | 0.06 | 0.42 | ± | 0.28 |
| RvD3 | 0.297 | ± | 0.10 | 0.12 | ± | 0.06 |
| RvD4 | 0.967 | ± | 0.38 | 1.17 | ± | 0.35 |
| RvD5 | 0.900 | ± | 0.29 | 0.61 | ± | 0.21 |
| RvD6 | 0.170 | ± | 0.10 | 0.14 | ± | 0.07 |
| 17R-RvD1 | 0.752 | ± | 0.36 | 0.40 | ± | 0.19 |
| 17R-RvD3 | 0.000 | ± | 0.00 | 0.01 | ± | 0.01 |
| PD1 | 0.378 | ± | 0.29 | 0.50 | ± | 0.27 |
| 17R-PD1 | 0.213 | ± | 0.16 | 0.09 | ± | 0.06 |
| 10S,17S-diHDHA | 1.539 | ± | 0.59 | 2.78 | ± | 1.25 |
| 22-OH-PD1 | 0.219 | ± | 0.14 | 0.10 | ± | 0.06 |
| PCTR1 | 13.344 | ± | 7.16 | 9.31 | ± | 3.38 |
| PCTR2 | 23.464 | ± | 16.33 | 17.25 | ± | 9.68 |
| PCTR3 | 144.870 | ± | 79.35 | 256.03 | ± | 108.45 |
| MaR1 | 3.396 | ± | 1.36 | 2.42 | ± | 0.43 |
| MaR2 | 0.604 | ± | 0.63 | 0.00 | ± | 0.00 |
| 22-OH-MaR1 | 1.931 | ± | 2.00 | 38.03 | ± | 13.01 |
| 22-COOH-MaR1 | 0.000 | ± | 0.00 | 0.00 | ± | 0.00 |
| 14-oxo-MaR1 | 0.067 | ± | 0.07 | 0.00 | ± | 0.00 |
| 7S,14S-diHDHA | 3.492 | ± | 1.05 | 1.82 | ± | 0.49 |
| 4,14-diHDHA | 0.367 | ± | 0.22 | 0.18 | ± | 0.15 |
| MCTR1 | 53.050 | ± | 23.37 | 19.11 | ± | 3.57 |
| MCTR2 | 15.557 | ± | 6.27 | 8.25 | ± | 4.01 |
| MCTR3 | 82.130 | ± | 44.27 | 165.40 | ± | 60.92 |
| **n-3 DPA Bioactive Metabolome** | | | | | | |
| RvT1 | 0.64 | ± | 0.20 | 0.74 | ± | 0.22 |
| RvT2 | 0.22 | ± | 0.15 | 0.54 | ± | 0.31 |
| RvT3 | 1.02 | ± | 0.57 | 0.28 | ± | 0.12 |
| RvT4 | 0.45 | ± | 0.28 | 0.44 | ± | 0.24 |
| RvD1_n-3 DPA_ | 0.06 | ± | 0.06 | 0.07 | ± | 0.04 |
| RvD2n-3DPA | 0.63 | ± | 0.33 | 0.98 | ± | 0.33 |
| RvD5_n-3DPA_ | 1.30 | ± | 0.52 | 1.58 | ± | 0.57 |
| PD1_n-3 DPA_ | 0.31 | ± | 0.12 | 0.14 | ± | 0.05 |
| PD2_n-3 DPA_ | 0.00 | ± | 0.00 | 0.00 | ± | 0.00 |
| 10S, 17S-diHDPA | 0.22 | ± | 0.09 | 0.15 | ± | 0.07 |
| 22-OH-PD1_n-3 DPA_ | 0.93 | ± | 0.69 | 0.13 | ± | 0.09 |
| MaR1n-3 DPA | 0.05 | ± | 0.05 | 0.18 | ± | 0.09 |
| MaR2_n-3 DPA_ | 32.02 | ± | 13.00 | 22.51 | ± | 2.78 |
| 7S,14S-diHDPA | 0.32 | ± | 0.18 | 0.19 | ± | 0.13 |
| **EPA Bioactive Metabolome** | | | | | | |
| RvE1 | 1.7311 | ± | 1.11 | 1.28 | ± | 0.42 |
| RvE2 | 0.6633 | ± | 0.69 | 19.39 | ± | 19.83 |
| RvE3 | 1.6686 | ± | 1.17 | 1.84 | ± | 0.64 |
| RvE4 | 67.7400 | ± | 15.03 | 139.50 | ± | 29.69 |
| **AA Bioactive Metabolome** | | | | | | |
| LXA_4_ | 0.43 | ± | 0.18 | 1.53 | ± | 0.71 |
| LXB_4_ | 1.36 | ± | 0.48 | 1.97 | ± | 0.98 |
| 5S,15S-diHETE | 9.63 | ± | 5.70 | 10.66 | ± | 2.86 |
| 15-epi-LXA_4_ | 2.16 | ± | 0.83 | 1.90 | ± | 0.44 |
| 15-epi-LXB_4_ | 13.47 | ± | 9.01 | 6.21 | ± | 1.67 |
| 13,14-dehydro-15-oxo-LXA_4_ | 0.03 | ± | 0.03 | 0.13 | ± | 0.06 |
| 15-oxo-LXA_4_ | 0.58 | ± | 0.37 | 0.49 | ± | 0.14 |
| LTB_4_ | 108.96 | ± | 41.27 | 23.27 | ± | 7.64 |
| 5S,12S-diHETE | 13.59 | ± | 4.67 | 7.69 | ± | 2.34 |
| 6-trans-LTB_4_ | 7.57 | ± | 2.69 | 1.71 | ± | 0.36 |
| 6-trans-12-epi LTB_4_ | 10.95 | ± | 3.52 | 3.60 | ± | 0.59 |
| 20-OH-LTB_4_ | 16.78 | ± | 10.21 | 1.00 | ± | 0.30 |
| 20-COOH-LTB_4_ | 13.84 | ± | 6.04 | 1.09 | ± | 0.31 |
| LTC_4_ | 67.70 | ± | 30.50 | 2.12 | ± | 1.28 |
| LTD_4_ | 19.64 | ± | 5.76 | 2.52 | ± | 0.52 |
| LTE_4_ | 82.71 | ± | 27.40 | 14.05 | ± | 4.23 |
| PGD_2_ | 7.90 | ± | 1.63 | 10.26 | ± | 3.13 |
| PGE_2_ | 17.32 | ± | 7.94 | 14.44 | ± | 4.46 |
| PGF_2α_ | 5.00 | ± | 1.40 | 7.58 | ± | 2.36 |
| TxB_2_ | 186.43 | ± | 101.55 | 15.12 | ± | 5.58 |

The table displays the mean values ± sem of LM concentrations in pg/mL from plasma collected from COVID-19 patients with severe disease (n=15) and critically ill patients (n=23).
