# Supplementary material for: Dysregulated plasma lipid mediator profiles in critically ill COVID-19 patients
Source: PLoS One. 2021 Aug 26;16(8):e0256226. doi: 10.1371/journal.pone.0256226 (PMC8389414; doi:10.1371/journal.pone.0256226)
Supplement: S2 Table — The table displays the mean values ± sem of LM concentrations in pg/mL from plasma collected from COVID-19 patients who were later discharged (n = 18) or did not survive the disease (n = 4). (DOCX) [file pone.0256226.s002.docx]

**S2 Table. Plasma LM Values from Survivors and Non-survivors Patients.**

| **Group** | **Survivors (n=18)** | | | **Non-survivors (n=4)** | | |
| --- | --- | --- | --- | --- | --- | --- |
|  | **mean** | **±** | **sem** | **mean** | **±** | **sem** |
| **DHA Bioactive Metabolome** | | | | | | |
| RvD1 | 0.236 | ± | 0.14 | 0.00 | ± | 0.00 |
| RvD2 | 0.486 | ± | 0.35 | 0.24 | ± | 0.27 |
| RvD3 | 0.143 | ± | 0.07 | 0.04 | ± | 0.05 |
| RvD4 | 1.269 | ± | 0.44 | 0.84 | ± | 0.33 |
| RvD5 | 0.785 | ± | 0.25 | 0.00 | ± | 0.00 |
| RvD6 | 0.151 | ± | 0.09 | 0.00 | ± | 0.00 |
| 17R-RvD1 | 0.403 | ± | 0.23 | 0.50 | ± | 0.35 |
| 17R-RvD3 | 0.017 | ± | 0.01 | 0.00 | ± | 0.00 |
| PD1 | 0.573 | ± | 0.34 | 0.18 | ± | 0.21 |
| 17R-PD1 | 0.098 | ± | 0.07 | 0.00 | ± | 0.00 |
| 10S,17S-diHDHA | 3.098 | ± | 1.61 | 1.70 | ± | 0.48 |
| 22-OH-PD1 | 0.123 | ± | 0.08 | 0.00 | ± | 0.00 |
| PCTR1 | 11.149 | ± | 4.20 | 3.37 | ± | 3.90 |
| PCTR2 | 20.972 | ± | 12.35 | 1.04 | ± | 0.80 |
| PCTR3 | 327.149 | ± | 135.08 | 0.00 | ± | 0.00 |
| MaR1 | 2.080 | ± | 0.51 | 4.01 | ± | 0.62 |
| MaR2 | 0.000 | ± | 0.00 | 0.00 | ± | 0.00 |
| 22-OH-MaR1 | 34.221 | ± | 14.18 | 20.05 | ± | 16.73 |
| 22-COOH-MaR1 | 0.000 | ± | 0.00 | 0.00 | ± | 0.00 |
| 14-oxo-MaR1 | 0.000 | ± | 0.00 | 0.00 | ± | 0.00 |
| 7S,14S-diHDHA | 1.670 | ± | 0.57 | 2.95 | ± | 1.29 |
| 4,14-diHDHA | 0.046 | ± | 0.05 | 0.80 | ± | 0.93 |
| MCTR1 | 19.900 | ± | 4.30 | 20.33 | ± | 7.09 |
| MCTR2 | 9.476 | ± | 5.12 | 2.36 | ± | 1.87 |
| MCTR3 | 211.341 | ± | 74.91 | 0.00 | ± | 0.00 |
| **n-3 DPA Bioactive Metabolome** | | | | | | |
| RvT1 | 0.68 | ± | 0.27 | 0.78 | ± | 0.51 |
| RvT2 | 0.70 | ± | 0.40 | 0.00 | ± | 0.00 |
| RvT3 | 0.31 | ± | 0.15 | 0.24 | ± | 0.16 |
| RvT4 | 0.45 | ± | 0.29 | 0.52 | ± | 0.61 |
| RvD1_n-3 DPA_ | 0.04 | ± | 0.03 | 0.21 | ± | 0.24 |
| RvD2n-3DPA | 0.83 | ± | 0.36 | 1.90 | ± | 1.11 |
| RvD5_n-3DPA_ | 1.69 | ± | 0.69 | 0.24 | ± | 0.27 |
| PD1_n-3 DPA_ | 0.11 | ± | 0.05 | 0.28 | ± | 0.21 |
| PD2_n-3 DPA_ | 0.00 | ± | 0.00 | 0.00 | ± | 0.00 |
| 10S, 17S-diHDPA | 0.20 | ± | 0.09 | 0.00 | ± | 0.00 |
| 22-OH-PD1_n-3 DPA_ | 0.16 | ± | 0.12 | 0.00 | ± | 0.00 |
| MaR1n-3 DPA | 0.23 | ± | 0.12 | 0.00 | ± | 0.00 |
| MaR2_n-3 DPA_ | 22.64 | ± | 3.49 | 23.39 | ± | 4.37 |
| 7S,14S-diHDPA | 0.25 | ± | 0.16 | 0.00 | ± | 0.00 |
| **EPA Bioactive Metabolome** | | | | | | |
| RvE1 | 1.1367 | ± | 0.50 | 1.32 | ± | 0.98 |
| RvE2 | 24.7797 | ± | 25.50 | 0.00 | ± | 0.00 |
| RvE3 | 1.9157 | ± | 0.78 | 1.93 | ± | 1.39 |
| RvE4 | 150.1533 | ± | 36.30 | 106.42 | ± | 63.29 |
| **AA Bioactive Metabolome** | | | | | | |
| LXA_4_ | 1.70 | ± | 0.91 | 1.08 | ± | 0.76 |
| LXB_4_ | 1.69 | ± | 1.19 | 3.73 | ± | 2.20 |
| 5S,15S-diHETE | 10.92 | ± | 3.51 | 6.66 | ± | 4.71 |
| 15-epi-LXA_4_ | 1.78 | ± | 0.52 | 2.94 | ± | 0.97 |
| 15-epi-LXB_4_ | 6.51 | ± | 2.10 | 3.83 | ± | 1.93 |
| 13,14-dehydro-15-oxo-LXA_4_ | 0.08 | ± | 0.06 | 0.36 | ± | 0.29 |
| 15-oxo-LXA_4_ | 0.36 | ± | 0.10 | 0.87 | ± | 0.82 |
| LTB_4_ | 21.56 | ± | 8.84 | 36.39 | ± | 22.22 |
| 5S,12S-diHETE | 7.03 | ± | 2.77 | 12.39 | ± | 5.77 |
| 6-trans-LTB_4_ | 1.40 | ± | 0.35 | 3.27 | ± | 1.33 |
| 6-trans-12-epi LTB_4_ | 3.40 | ± | 0.68 | 5.10 | ± | 1.28 |
| 20-OH-LTB_4_ | 0.70 | ± | 0.26 | 2.47 | ± | 1.27 |
| 20-COOH-LTB_4_ | 1.11 | ± | 0.36 | 1.28 | ± | 0.86 |
| LTC_4_ | 2.71 | ± | 1.63 | 0.00 | ± | 0.00 |
| LTD_4_ | 2.49 | ± | 0.62 | 2.97 | ± | 1.39 |
| LTE_4_ | 15.07 | ± | 5.39 | 12.35 | ± | 3.87 |
| PGD_2_ | 9.87 | ± | 3.72 | 14.25 | ± | 8.13 |
| PGE_2_ | 10.15 | ± | 3.07 | 36.48 | ± | 22.42 |
| PGF_2α_ | 7.52 | ± | 2.78 | 8.91 | ± | 7.00 |
| TxB_2_ | 14.91 | ± | 6.89 | 19.84 | ± | 10.74 |

The table displays the mean values ± sem of LM concentrations in pg/mL from plasma collected from COVID-19 patients who were later discharged (n=18) or did not survive the disease (n=4).
