# Supplementary material for: Dysregulated plasma lipid mediator profiles in critically ill COVID-19 patients
Source: PLoS One. 2021 Aug 26;16(8):e0256226. doi: 10.1371/journal.pone.0256226 (PMC8389414; doi:10.1371/journal.pone.0256226)
Supplement: Attachment — Submitted filename: Palmas, Clarke et al response to reviewer comments.docx [file pone.0256226.s003.docx]

**Comments to the Author**

Reviewer #1: This is a well-written and well-organized submission that describes a role for lipid mediators, particularly special proresolving mediators (SPMs) in the pathogenesis of SARS CoV2 infection, and the potential of using these mediators for prediction of disease outcome. The data are clear and well-presented and the conclusions of the study well-supported. The only aspect that could be improved is the description of the trial design itself in the methods. It would make the paper much clearer if the experimental design was described in some detail.

*We would like to thank the reviewer for their positive comments. We agree with your comments about the study design and a more comprehensive description of the study methods has now been added. We have also amended a numerical error in the WHO scores, which was noticed upon review.*

Reviewer #2: This is an outstanding scientific research report from an internationally recognized group of investigators. They report on the levels of specialized pro-resolving and pro-inflammatory lipid mediators in two groups of subjects with Covid-19: critically ill (admitted to ICU) and severe disease (hospitalized but not in ICU) using state-of-the-art liquid chromatography mass spectrometry. They found that plasma concentrations of both pro-inflammatory and pro-resolving lipid mediators were lower in critically ill patients compared to those with severe disease. Levels were lowest in those who died. Their findings establish a link between plasma lipid mediators, disease severity and survival in Covid-19. The findings have important implications for etiology of severe versus critically ill patients with Covid-19 and treatment. I have only a few minor comments:

line 260: "liked" should be "linked"

line 284: "the" should be "they"

line 285: Sentence starting with "while" is not a complete sentence

line 307: macrophage should be pleural

line 308: deleted "note"

Table 2: I believe this is for critically ill rather than severe patients. Severe is noted in the header.

line 566: 'ration' should be "ratio"

Table S1: It appears that the header "moderate" should be "severe" and the heading severe should be critically ill.

*We would like to thank the reviewer for their kind comments and the recognition of significance of our hard. Moreover, we would like to express our appreciation for the detailed feedback provided, which allowed us to polish the delivery of this manuscript.*

*We corrected the typos highlighted in your comment.*
